# Supplementary material for: Exploring the antioxidant potential of chalcogen-indolizines throughout in vitro assays
Source: PeerJ. 2024 Mar 15;12:e17074. doi: 10.7717/peerj.17074 (PMC10946399; doi:10.7717/peerj.17074)

**Supplementary Material**

**Exploring the Antioxidant Potential of Chalcogen-indolizines throughout *in vitro* assays**

Cleisson S. Garcia^1^, Marcia J. da Rocha^1^, Marcelo H. Presa^1^, Camila S. Pires^1^, Evelyn M. Besckow^1^, Filipe Penteado^2^, Caroline S. Gomes^3^, Eder J. Lenardão^3^, Cristiani F. Bortolatto^1^ and César A. Brüning^1^

^1^ Laboratory of Biochemistry and Molecular Neuropharmacology (LABIONEM), Graduate Program in Biochemistry and Bioprospecting (PPGBBio), Chemical, Pharmaceutical and Food Sciences Center (CCQFA), Federal University of Pelotas (UFPel), P.O. Box, 354, 96010-900, Pelotas, RS, Brazil.

^2^ Department of Chemistry, Federal University of Santa Maria (UFSM), Av. Roraima, Building 18, 97105-340, Santa Maria, RS, Brazil.

^3^ Laboratory of Clean Organic Synthesis (LASOL), Chemical, Pharmaceutical and Food Sciences Center (CCQFA), Federal University of Pelotas (UFPel), P.O. Box, 354, 96010-900, Pelotas, RS, Brazil.

2-phenyl-1-(phenylthio)indolizine (**SIN-1**)

^1^H NMR (CDCl_3_, 400 MHz) *δ* (ppm) 7.86 (d, *J* = 6.9 Hz, 1H); 7.58 – 7.51 (m, 2H); 7.51 – 7.41 (m, 2H); 7.29 – 7.21 (m, 2H); 7.20 – 7.14 (m, 1H); 7.08 – 7.00 (m, 2H); 6.96 – 6.88 (m, 3H); 6.71 (ddd, *J* = 9.0, 6.6, 0.8 Hz, 1H); 6.54 – 6.46 (m, 1H). ^13^C NMR (CDCl_3_, 100 MHz) *δ* (ppm) 140.6, 137.4, 134.2, 133.2, 128.7, 128.3, 127.0, 125.5, 125.1, 124.4, 119.7, 118.0, 111.9, 111.7, 94.6.

2-(4-chlorophenyl)-1-(phenylthio)indolizine (**SIN-2**)

^1^H NMR (CDCl_3_, 400 MHz) *δ* (ppm) 7.87 (d, *J* = 6.8 Hz, 1H); 7.53 – 7.44 (m, 4H); 7.21 (d, *J* = 8.5 Hz, 2H); 7.09 – 7.02 (m, 2H); 6.97 – 6.86 (m, 3H); 6.77 – 6.70 (m, 1H); 6.57 – 6.49 (m, 1H). ^13^C NMR (CDCl_3_, 100 MHz) *δ* (ppm) 140.3, 137.6, 133.0, 132.6, 131.9, 129.9, 128.8, 128.5, 125.5, 125.1, 124.5, 120.0, 118.0, 112.1, 111.7, 94.6.

1-(phenylthio)-2-(p-tolyl)indolizine (**SIN-3**)

^1^H NMR (CDCl_3_, 400 MHz) *δ* (ppm) 7.86 (d, *J* = 6.7 Hz, 1H); 7.52 – 7.38 (m, 4H); 7.10 – 7.00 (m, 4H); 6.96 – 6.87 (m, 3H); 6.74 – 6.67 (m, 1H); 6.54 – 6.45 (m, 1H); 2.25 (s, 3H). ^13^C NMR (CDCl_3_, 100 MHz) *δ* (ppm) 140.7, 137.4, 136.7, 133.3, 131.2, 129.1, 128.7, 128.5, 125.5, 125.1, 124.3, 119.6, 117.9, 111.8, 111.6, 94.5, 21.1.

2-(4-chlorophenyl)-1-((4-fluorophenyl)thio)indolizine (**SIN-4**)

^1^H NMR (CDCl_3_, 400 MHz) *δ* (ppm) 7.89 (d, *J* = 6.9 Hz, 1H); 7.53 – 7.43 (m, 4H); 7.24 (d, *J* = 8.5 Hz, 2H); 6.89 – 6.81 (m, 2H); 6.80 – 6.72 (m, 3H); 6.58 – 6.52 (m, 1H). ^13^C NMR (CDCl_3_, 100 MHz) *δ* (ppm) 160.7 (d, *J* = 243.7 Hz), 137.5, 135.2 (d, *J* = 2.9 Hz), 133.1, 132.6, 131.8, 129.9, 128.5, 126.9 (d, *J* = 7.7 Hz), 125.6, 120.1, 117.9, 115.8 (d, *J* = 22.1 Hz), 112.1, 111.8, 95.1.

2-(4-chlorophenyl)-1-((4-methoxyphenyl)thio)indolizine (**SIN-5**)

^1^H NMR (CDCl_3_, 400 MHz) *δ* (ppm) 7.85 (d, *J* = 6.9 Hz, 1H); 7.56 – 7.47 (m, 3H); 7.43 (s, 1H); 7.23 (d, *J* = 8.5 Hz, 2H); 6.87 (d, *J* = 8.8 Hz, 2H); 6.78 – 6.71 (m, 1H); 6.63 (d, *J* = 8.8 Hz, 2H); 6.55 – 6.49 (m, 1H); 3.63 (s, 3H). ^13^C NMR (CDCl_3_, 100 MHz) *δ* (ppm) 157.5, 137.4, 132.9, 132.8, 131.7, 130.9, 129.9, 128.5, 127.2, 125.5, 119.8, 118.0, 114.5, 112.0, 111.5, 96.2, 55.3.

2-(4-chlorophenyl)-1-(phenylselanyl)indolizine (**ClSeI**)

^1^H NMR (CDCl_3_, 400 MHz) *δ* (ppm) 7.92 (d, *J* = 6.9 Hz, 1H); 7.59 (d, *J* = 9.0 Hz, 1H); 7.54-7.47 (m, 3H); 7.29 (d, *J* = 8.4 Hz, 2H); 7.13-7.01 (m, 5H); 6.83-6.75 (m, 1H); 6.61-6.54f (m, 1H). ^13^C NMR (CDCl_3_, 100 MHz) *δ* (ppm) 137.7, 135.0, 133.3, 132.9, 132.4, 130.2, 129.0, 128.3, 127.8, 125.4, 119.8, 119.0, 111.9, 91.5.

1-(phenylselanyl)-2-(p-tolyl)indolizine (**MeSeI**)

^1^H NMR (CDCl_3_, 400 MHz) *δ* (ppm) 7.86 (d, *J* = 6.7 Hz, 1H); 7.52 – 7.38 (m, 4H); 7.10 – 7.00 (m, 4H); 6.96 – 6.87 (m, 3H); 6.74 – 6.67 (m, 1H); 6.54 – 6.45 (m, 1H); 2.25 (s, 3H). ^13^C NMR (CDCl_3_, 100 MHz) *δ* (ppm) 140.7, 137.4, 136.7, 133.3, 131.2, 129.1, 128.7, 128.5, 125.5, 125.1, 124.3, 119.6, 117.9, 111.8, 111.6, 94.5, 21.1.

2-phenyl-1-(phenylselanyl)indolizine (**SeI**)

^1^H NMR (CDCl_3_, 400 MHz) *δ* (ppm) 7.86 (d, *J* = 6.9 Hz, 1H); 7.56 – 7.54 (m, 2H); 7.49 – 7.47 (m, 2H); 7.27 – 7.23 (m, 2H); 7.19 – 7.15 (m, 1H); 7.07 – 7.03 (m, 2H); 6.94 – 6.91 (m, 3H); 6.72 (ddd, *J* = 9.0, 6.6, 0.8 Hz, 1H); 6.51 (td, *J* = 6.9, 1.0 Hz, 1H). ^13^C NMR (CDCl_3_, 100 MHz) *δ* (ppm) 137.6, 135.3, 134.8, 133.7, 129.1, 128.9, 128.2, 127.9, 127.0, 125.4, 125.2, 119.6, 119.1, 112.0, 111.8, 91.6.


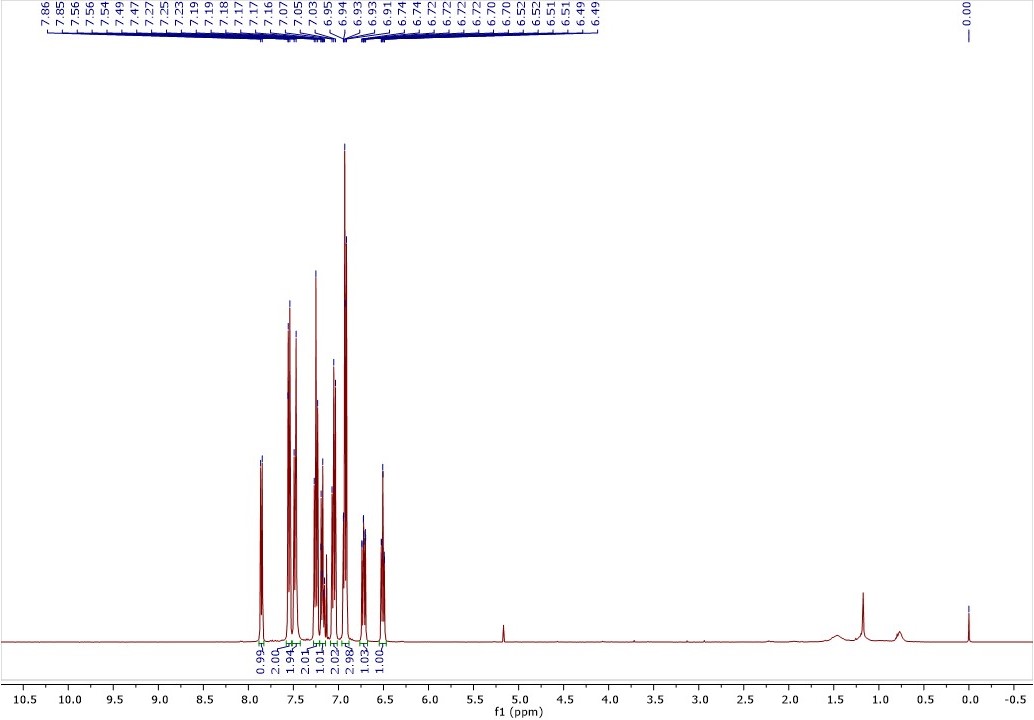


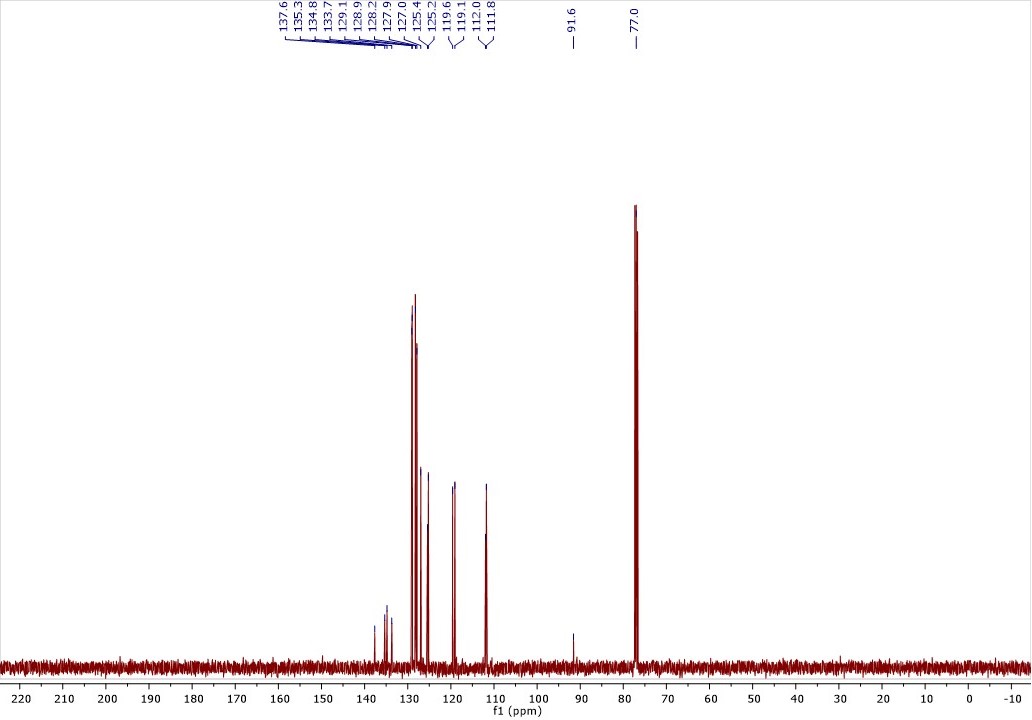

Supplement: Supplemental Information 1 [file peerj-12-17074-s001.docx]
